# Scleromyxedema Managed With High‐Dose Intravenous Immunoglobulin and Bortezomib–Dexamethasone: A Case Report

**DOI:** 10.1155/crom/3773160

**Published:** 2026-02-26

**Authors:** Darren Wijaya, Zachary Hanson, Eric K. Lau, Mojtaba Akhtari

**Affiliations:** ^1^ Department of Internal Medicine, Loma Linda University Health, Loma Linda, California, USA, lluh.org; ^2^ Division of Medical Oncology and Hematology, Loma Linda University Health, Loma Linda, California, USA, lluh.org; ^3^ Palo Alto Medical Foundation, Sutter Health, Palo Alto, California, USA, sutterhealth.org

## Abstract

Scleromyxedema is a rare, chronic cutaneous mucinosis marked by widespread waxy papules and potential extracutaneous involvement. This case report discusses the management of a 48‐year‐old female diagnosed with scleromyxedema, who initially partially responded to high‐dose intravenous immunoglobulin (HDIVIG) therapy. After partial relapse, she was induced with bortezomib, a proteasome inhibitor, and dexamethasone, achieving significant clinical improvement. Long‐term maintenance with IVIG was utilized to prevent recurrence of symptoms. This case highlights the effectiveness of both HDIVIG and bortezomib–dexamethasone dual therapy as viable treatment options for scleromyxedema, emphasizing the importance of maintenance therapy to prevent relapse.

## 1. Introduction

Scleromyxedema is an exceptionally rare, chronic cutaneous mucinosis with a prevalence of less than 1 in 1,000,000 adult patients [1, 2]. It is characterized by widespread, firm, waxy papules measuring 2–3 mm in diameter, often arranged linearly or in clusters and coalescing into indurated, sclerodermoid plaques [3]. Lesions are typically distributed symmetrically on the face, neck, upper trunk, and extremities, leading to progressive skin thickening, reduced mobility, and occasionally leonine facies [3]. Extracutaneous manifestations are not uncommon and can be significant or even life‐threatening. Neurologic complications often include peripheral neuropathy, with some cases progressing to “dermatoneuro syndrome (DNS)” characterized by acute encephalopathy [4]. Cardiac involvement varies from arrhythmias and pericarditis to heart failure due to myocardial infiltration or fibrosis [3]. Rheumatologic features often resemble systemic sclerosis, presenting as joint stiffness, arthritis, or tendon involvement [3]. Additionally, gastrointestinal, pulmonary, and renal systems may occasionally be affected, further complicating the disease course [3]. “Severe” scleromyxedema is commonly defined by the presence of life‐threatening complications such as DNS or cardiomyopathy [5].

While the precise pathogenesis is unknown, scleromyxedema is typically associated with monoclonal gammopathies [1]. Additionally, increased levels of circulating cytokines such as interleukin‐1 (IL‐1), tumor necrosis factor‐alpha (TNF‐*α*), and transforming growth factor‐beta (TGF‐*β*) have been noted, which are known to stimulate glycosaminoglycan synthesis and fibroblast proliferation in the skin [6]. Ultimately, histopathology has identified a triad of microscopic features: Diffuse mucin deposits in the upper and mid–reticular dermis, increased collagen deposition, and marked proliferation of irregularly arranged fibroblasts [7].

The approach to the management of scleromyxedema is mainly based on case reports and case series, as no randomized trials have been conducted. Treatment typically involves high‐dose intravenous immunoglobulin (HDIVIG) at 2 g/kg/month and anti–plasma cell therapy. Successful treatment is considered achieving at least a clinical partial response (PR) if not a clinical complete response (CR). Paraprotein levels do not have to normalize in order to achieve clinic response and do not correlate with disease severity, progression, or treatment response [3]. Even after achieving an initial response, patients typically have to continue on maintenance therapy with IVIG and/or anti–plasma cell therapy.

This case report discusses a patient with scleromyxedema in the setting of monoclonal gammopathy who was successfully treated with HDIVIG and bortezomib–dexamethasone dual therapy.

## 2. Case Presentation

### 2.1. Patient Information

The patient is a 48‐year‐old female with a medical history of asthma, gastric sleeve procedure in March 2015, diverticulitis, and cervical cancer in 2002, who initially presented in March 2020 to a community dermatology clinic with a chief complaint of diffuse rash and hair loss.

### 2.2. Presentation

**Figure 1:**
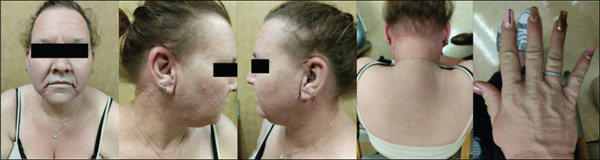
Waxy, skin‐colored papules at initial presentation involving the forehead, cheeks, nape of the neck, and dorsal hands.

The patient noted that her skin lesions first appeared in January 2020. Dermatology described numerous skin‐colored papules coalescing into plaques on the scalp, forehead, cheeks, nape of the neck, chest, axillae, and breasts (Figure 1). On the arms, the lesions involved the extensor surfaces, and on the legs, she had folliculocentric papules. There was no involvement of the eyelids, lips, oral mucosa, nails, or digits. She also reported reduced handgrip strength due to tightening of the skin over the dorsal aspects of all fingers and both hands.

Laboratory studies showed mild normocytic anemia (hemoglobin 11.8 g/dL, MCV 95), low serum albumin (3.0 g/dL), and low total protein (5.8 g/dL). The remainder of the CBC and CMP were unremarkable. Additional testing, including TSH, total T4, zinc, ferritin, vitamin D 25‐OH, vitamin B12, total and free testosterone, ANA, ESR, and CRP, was also within normal limits.

Despite treatment with topical steroids, the skin lesions persisted for several months. Initially nonpainful and nonpruritic, they gradually became severely pruritic and were associated with diffuse joint and body pain, worst in her hips and knees.

**Figure 2:**
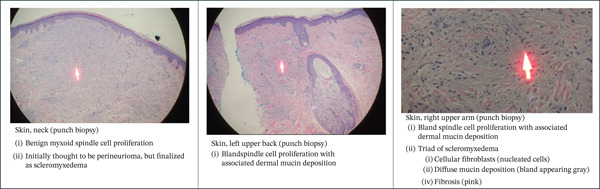
Histopathological analysis of skin lesion samples.

A biopsy was first taken in June 2020 from a pearly lesion on her neck. It was described as a benign myxoid spindle cell proliferation (Figure 2). Immunohistochemical stains were positive for CD34 and faint, patchy, focal positive staining on the EMA stain. Tumor cells stains were negative for S100 protein, NSE, GFAP, CD57, SMA, Factor XIIIa, CK5/6, and AE1/3. Given the histologic and immunohistochemical features, the diagnosis of perineuroma was initially favored.

However, other punch biopsies in October 2020 from the left upper back and right upper arm (each 4 mm in diameter) revealed bland spindle cell proliferation with associated dermal mucin deposition, consistent with either papular mucinosis or scleromyxedema (Figure 2). Follow‐up serum protein electrophoresis revealed a monoclonal protein peak (M spike) of 0.7 g/dL (10.7%), but no immunofixation was performed. There was insufficient protein to produce a detectable pattern on urine protein electrophoresis. A CT‐guided bone marrow biopsy showed a small (0.4%) population of lambda‐restricted clonal plasma cells, which were identified via flow cytometry. The karyotype was normal (46,XX [8]).

Given the histopathology findings and presence of monoclonal gammopathy, she was diagnosed with scleromyxedema and began treatment in February 2021.

### 2.3. Treatment Course

**Figure 3:**
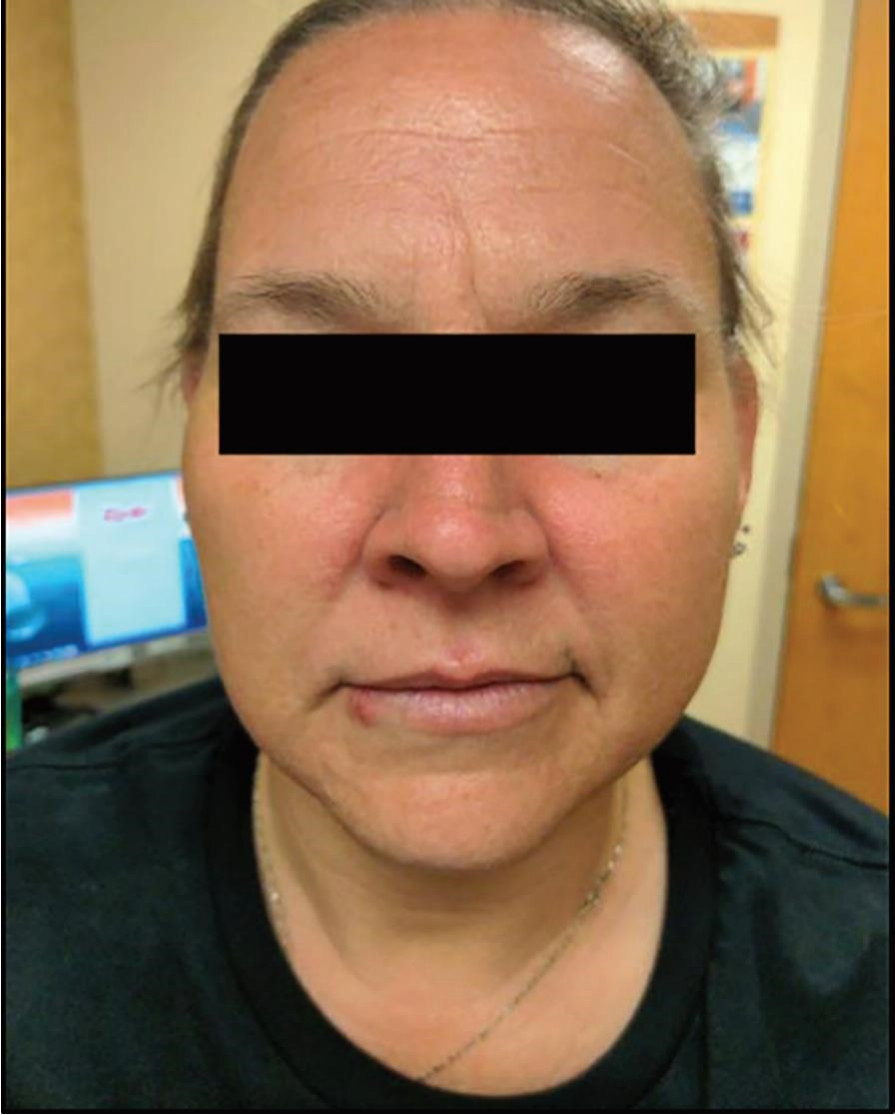
Waxy, skin‐colored papules on the forehead and cheeks after six cycles of HDIVIG.

In February 2021, the patient began treatment with HDIVIG at a dose of 2 g/kg/month for a planned six cycles. Within 2 months, she experienced a noticeable improvement in her cutaneous symptoms and completed initial therapy by July 2021 with a very good partial clinical response (Figure 3), characterized by significant regression of waxy papules throughout the body and reduced skin thickening of the hands. Her pruritus was well‐controlled with gabapentin.

Subsequently, she transitioned to maintenance therapy with IVIG at 1 g/kg every other month. By the second maintenance dose, her skin abnormalities began to return, and she developed new dysphonia and peripheral neuropathy.

Consequently, from November 2021 to January 2022, she underwent induction with subcutaneous bortezomib, a proteasome inhibitor (1.3 mg/m^2^, up to 2 mg), and dexamethasone (40 mg weekly). Following improvements in her dysphonia and skin condition, along with the development of likely bortezomib‐related injection‐site reactions, headaches, and severe constipation, it was decided that she had derived significant benefit from the bortezomib and dexamethasone regimen. To avoid further side effects, this therapy was discontinued after 12 weeks, and she restarted on maintenance therapy with HDIVIG (2 g/kg monthly). She continues to tolerate HDIVIG, with her cutaneous symptoms and dysphonia maintaining excellent PR to date (32 months after completing bortezomib + dexamethasone therapy). However, her peripheral neuropathy and arthralgias persist, with multiple rheumatologists attributing her pain to scleromyxedema.

## 3. Discussion

Scleromyxedema is a rare MGCS for which no established treatment guidelines exist. Treatment options are available, but evidence of efficacy is limited to retrospective studies and case reports. Notably, IVIG and anti–plasma cell–directed medications are popular choices for initial treatment as well as maintenance therapy. Successful treatment is defined as achieving a clinical CR or at least PR, regardless of paraprotein levels, which do not correlate with disease severity, progression, or response [3].

Our patient demonstrated an excellent PR to initial HDIVIG monotherapy, aligning with findings from other literature. A retrospective multicenter study published in *Blood* evaluated clinical responses to different treatment regimens in patients with scleromyxedema, irrespective of hematologic paraprotein response [5]. We performed an informal secondary analysis of this study and found that among 25 patients treated with HDIVIG alone, 13 achieved clinical CR and 12 had PR. In comparison, 10 patients who received combination HDIVIG therapy with corticosteroids achieved two CRs and eight PRs. While the sample size was limited, this observation suggests that both approaches may offer comparable efficacy. Additionally, a prospective open‐label study demonstrated significant improvement in eight patients with scleromyxedema treated with HDIVIG alone, showing an 81.6% reduction in mRSSS (modified Rodnan score system for scleromyxedema) and no major side effects [9].

In both the retrospective and prospective studies, most patients required maintenance therapy to manage their disease [5, 9]. In the retrospective study, seven out of eight patients relapsed after discontinuing HDIVIG for more than 3 months, with a median relapse time of 13 months (ranging from 1 to 24 months). Similarly, in a prospective study, six patients required ongoing maintenance HDIVIG infusions, and the two who stopped treatment relapsed after 6 and 25 months, respectively. In all instances, the maintenance dose was 2 g/kg every 4–6 weeks. Other studies also show consistent success with HDIVIG maintenance, and all attempts to discontinue IVIG have led to a gradual recurrence of skin lesions [10, 11]. To date, no documented cases have demonstrated success with reduced dosages, suggesting that 2 g/kg every 4–6 weeks is an optimal maintenance dose and reducing the dosage may increase the risk of relapse. Our own attempt to lower the dose to 1 g/kg every 8 weeks further supports this finding. IVIG is generally considered safe, with side effects typically occurring during the first infusion or when switching to a new brand [12]. However, patients receiving regular IVIG infusions rarely experience such adverse effects [13–15]. The primary challenges with long‐term IVIG therapy are the high cost and the inconvenience of frequent infusions.

We used an anti–plasma cell therapy consisting of bortezomib and dexamethasone for induction after initial relapse. This treatment led to clinical PR, with improvement in cutaneous symptoms and dysphonia, though arthralgias and peripheral neuropathy persisted. To date, only five documented cases have used this two‐drug regimen for induction [5, 16–19] (Table 1). Among these, four patients achieved clinical CR, and one achieved PR. Additionally, three patients reached serologic CR. Notably, two of these patients presented with severe manifestations, including DNS and ischemic cardiomyopathy, all of which completely resolved. Separately, one reported case described a patient who failed HDIVIG and relapsed 9 months after autologous stem cell transplantation with melphalan, but subsequently achieved a sustained complete clinical and hematologic response to bortezomib–dexamethasone lasting at least 10 months [17]. Taken together, these results suggest that dual therapy with bortezomib and dexamethasone may be a highly effective induction strategy for both severe and nonsevere forms of the disease. Other bortezomib‐based regimens often include a third drug, such as an immunomodulator or IVIG [5, 8, 20, 21]. While these combinations may be effective, they also come with a higher risk of side effects and increased costs. In contrast, bortezomib monotherapy may be less effective than when combined with dexamethasone, although one study did report a PR to bortezomib used alone [10].

**Table 1:** Summary of five cases of induction therapy with bortezomib and dexamethasone dual therapy.

Patient	Distribution of disease	Regimen	Prior treatments (reason stopped)	Response
1 [5]	Skin, DNS, epilepsy, and ischemic cardiopathy	Bortezomib, 1.3 mg/m^2^ IVP on Days 1, 4, 8, and 11Dexamethasone, 40 mg on Days 1, 2, 3, 4, 8, 9, 10, and 11, for 4 months	• None	• Clinical CR
2 [16]	Skin, arthralgias	Bortezomib, 1.3 mg/m^2^ on Days 1, 4, 8, and 11Dexamethasone, 40 mg on Days 1–4, every 21 days, for eight cycles	• Methotrexate (refractory)• Melphalan + low‐dose methylprednisolone (refractory)• Lenalidomide + dexamethasone (refractory)	• Rapid improvement of skin and arthralgias within the first cycle and continued improvement after completing therapy. Clinical CR in 24 months after initiation• Serologic CR
3 [17]	Skin, peripheral edema, poor mouth/tongue motility (dysphagia)	Bortezomib, 1.3 mg/m^2^ on Days 1, 4, 8, and 11Dexamethasone, 20 mg/day on Days 1, 2, 4, 5, 8, 9, 11, and 12, in 21‐day courses	• Prednisone (refractory)• IVIG 1 g/kg/day × 2 consecutive days (single course; refractory)• Bone marrow transplant + melphalan (successful induction, but relapse 9 months later)	• Clinical CR• Serologic CR
4 [18]	Skin, DNS, peripheral neuropathy, esophageal peristalsis (dysphagia)	Bortezomib + dexamethasone for 8 months followed by bortezomib monotherapy for an additional 4 months	• None	• Resolution of skin symptoms by Month 1. Clinical CR by Month 8• Serologic CR by Month 8
5 [19]	Skin	Bortezomib + dexamethasone on Days 1, 4, 8, and 11, repeated every 21 days	• Prednisolone + hydroxychloroquine (refractory)	• Clinical PR: Cutaneous lesions have mostly subsided after four cycles

Regarding the treatment of severe scleromyxedema, there is a significant lack of evidence, including case reports, to reliably compare the efficacy of HDIVIG, anti–plasma cell therapy, corticosteroids, plasmapheresis, or their combinations. However, due to the availability of more evidence supporting the successful treatment of HDIVIG‐refractory cases with anti–plasma cell therapy and plasmapheresis, it is generally accepted to prioritize anti–plasma cell therapy and corticosteroids for severe manifestations like DNS and cardiomyopathy [5, 22]. This approach is justified by the urgency of these complications, where rapid and effective treatment is essential. In addition, autologous stem cell transplantation has shown preliminary potential as a treatment option with good efficacy and safety [23–27].

## 4. Conclusion

This case highlights the effectiveness of both HDIVIG and bortezomib–dexamethasone dual therapy as viable induction treatment options for scleromyxedema. Our patient′s PR to initial HDIVIG monotherapy aligns with findings from previous studies, while induction with bortezomib and dexamethasone offers a promising alternative salvage approach for HDIVIG‐refractory disease. Long‐term maintenance therapy with IVIG is crucial, as reducing or discontinuing the dosage led to relapse in both our patient and other reported cases. Future research should focus on clinical trials to further refine and optimize treatment and maintenance strategies for managing scleromyxedema.

## Funding

No funding was received for this manuscript.

## Disclosure

The institution provided support solely for publication costs and was not involved in the study′s design, writing, or decision to publish.

## Consent

Written informed consent was obtained from the patient for publication of this case report, in accordance with the International Committee of Medical Journal Editors (ICMJE) recommendations.

## Conflicts of Interest

The authors declare no conflicts of interest.

## Data Availability

The data that support the findings of this study are available in the *Blood* journal at 10.1182/blood.2019002300, Reference Number: *Blood*. 2020; 135(14): 1101–1110. These data were derived from the following resource available in the public domain: *Blood* journal, doi:10.1182/blood.2019002300.
